# Study of the cross-market effects of Brexit based on the improved symbolic transfer entropy GARCH model—An empirical analysis of stock–bond correlations

**DOI:** 10.1371/journal.pone.0183194

**Published:** 2017-08-17

**Authors:** Xiurong Chen, Yixiang Tian, Rubo Zhao

**Affiliations:** School of Management and Economics, University of Electronic Science and Technology of China, Chengdu, China; East China University of Science and Technology, CHINA

## Abstract

In this paper, we study the cross-market effects of Brexit on the stock and bond markets of nine major countries in the world. By incorporating information theory, we introduce the time-varying impact weights based on symbolic transfer entropy to improve the traditional GARCH model. The empirical results show that under the influence of Brexit, flight-to-quality not only commonly occurs between the stocks and bonds of each country but also simultaneously occurs among different countries. We also find that the accuracy of the time-varying symbolic transfer entropy GARCH model proposed in this paper has been improved compared to the traditional GARCH model, which indicates that it has a certain practical application value.

## Introduction

Investors often hold multiple assets to effectively reduce the risk of loss during a financial crisis. It is typically believed that cross-market risk contagion is caused in crisis periods when the diversification of assets fails to provide obvious benefits. This phenomenon is often associated with financial contagion across stock markets. Conversely, it is typically believed that cross-market flight is induced when the prices of certain assets increase during a financial crisis, this phenomenon often occurs between the stock and bond markets in crisis periods. And it consists of two different behaviors. One is called flight-to-quality if investors buy bonds when sell stocks. The other is called flight-from-quality if investors buy bonds when sell stocks. So if the co-movement of stock and bond markets turn negative in a financial crisis, the investors could suffer less loss by holding both assets since one of which will provide positive returns. This indicates that cross-market flights including flight-to-quality from stocks to bonds and flight-from-quality from bonds to stocks can potentially increase the stability and resiliency of financial system because of the function of helping investors suffer less losses in financial crises. And cross-market risk contagion and cross-market flight are collectively called cross-market effects.

There have been numerous research findings on risk contagion and flight. Keim and Stambaugh conducted research on the correlation between the stock market and the bond market in 1986 for the first time[1]. A positive correlation is found in the returns of the stock market and the bond market during 1929–2001 in the United States according to the research conducted by Ilmanen; however, there is negative correlation during certain sub-sample periods. Additionally, the researcher attributed the factors affecting the correlation between the two asset classes to economic currency policy cycle, inflation and volatility shocks [2]. King and Wadhwani analyzed the phenomenon that nearly all stock markets in different economies declined despite the large differences in economic environments during October 1987, and they verified the existence of risk contagion effects among the stock markets of different countries by constructing models [3]. According to the research on sovereign risk contagion in the Eurozone during 2008–2012 conducted by Meitiu, there were obvious risk contagion effects on the long-term bond yields among countries in the Eurozone during that period. In addition, it was detected that the market risk preference was the important decisive factor for the sovereign risk [4]. Gai and Kapadia analyzed the influences of network structure change and capital market liquidity on the contagion probability by constructing a financial network analysis model in an arbitrary structure and discovered that the financial system has a robust but fragile feature; when the contagion probability is low but a crisis occurs, the contagion effects remain very wide [5]. According to the research on the correlation between the stocks and the bonds of 8 global economic entities during financial crisis events, Baur and Lucey discovered that the flights phenomena widely existed among economic entities, and there was an internal relationship between flights and risk contagions [6]. According to research on the correlation of stocks and bonds before, during and after the global financial crisis that originated in the United States, by utilizing DCC model, Mustafa and Samsudin verified the existence of flight behaviors of investors from the stock market to the bond market [7].

With the increasing deepening of global integration and financial liberalization, the relations among countries, regions and cities are also becoming increasingly closer. The economic crisis or emergency of any economic entity may lead to “domino” effects, affecting neighborhood or economic entities with close trade relations, then resulting in cross-market risk contagion or flight effect, which leads to market fluctuation or influence on the global economy. Especially since the 1980s, global financial crises have erupted unceasingly, such as the debt crisis in Latin America in the 1980s, the financial crisis in Mexico in 1994, the “9/11” event in the United States in 2001, the financial crisis in Argentina in 2002, the American subprime mortgage crisis during 2007–2009 and the European sovereign debt crisis during 2010–2012, all of which caused major fluctuations in global financial markets. Therefore, the study on cross-market effects in financial markets has increasingly practical significance; this also plays a guiding role for investors and policy makers, garnering the focus of numerous scholars. The year 2016 can be described as a year of global “black swan events”. First, Britain conducted public voting regarding breaking away from the EU on June 23, ending with approval. As the first black swan event, this led to significant impacts on the European financial markets and the global financial markets; the stocks of various main economic entities declined in a short period, and the pound and the Euro devaluated, while the U.S. dollar and the Japanese yen appreciated. Trump was selected as the 45^th^ president of the US after a political campaign against Hillary Clinton on November 8^;^ as the second black swan event in that year, it has led to influences on political patterns as well as global economy and diplomacy, and the economic policy advocated by Trump will have great significance on the global economy. Then, Italy conducted public voting for a constitutional amendment on December 4, which failed the next day due to the majority of votes against it. In addition, the prime minister of Italy announced his resignation on the same day; this was the third black swan event of 2016. The euro experienced a sharp decline after the publishing of the result. There may be huge trouble for the stability of the Euro financial system, and Italy is considered to exhibit the trend of breaking away from the EU.

We used the first black swan event, the British referendum of leaving the EU (Hereafter, Brexit) of 2016, as the background. We also utilize the research on the cross-market effects on the stocks and the bonds by Baur and Lucey through the utilization of the traditional GARCH model [6]. In this paper, by incorporating information theory, we construct the improved GARCH model based on the time-varying symbolic transfer entropy to conduct research on the cross-market risk contagion and flights in the stocks and bonds of 9 major global economies caused by Brexit. At the same time, we also conduct research on the simultaneity of the cross-market effects among various economies by using the panel GARCH model based on time-varying symbolic transfer entropy.

## Theory analysis and modeling

### 2.1 Definition

Generally, the cross-market effect is regarded as the cross-market flight phenomenon and the cross-market risk contagion phenomenon caused by financial risk.

The financial risks spread among different financial sub-markets, such as between stocks and bonds or between the foreign exchange markets and stocks, etc. The cross-market risk contagion is generally defined as the phenomenon of the significant positive increase in the correlation between financial markets during economic crisis periods compared to that of non-crisis period [6,8–10]. That is, when the degree of co-movement in the same direction between different markets is increased, it is considered that the cross-market risk contagion phenomenon occurs between the two markets. For example, the correlation changes from the negative before a crisis to the positive during a crisis, or there is a positive increase in the correlation during a crisis compared to the positive correlation before a crisis. However, if the positive increase only exists, the correlation between two markets remains negative; thus, it is not the risk contagion phenomenon. For example, the correlation changes from -0.8 to -0.3; although there is a positive increase of 0.5, it cannot be considered that contagion occurs, as the two markets continue to move in different directions.

Conversely, the investors would flee from one market into others if there exist sub-financial markets whose prices are increasing while other markets are negatively impacted by crises. The corresponding flight is often defined as the phenomenon of significant negative decrease in correlations between markets in crisis periods than that in non-crisis period [6,8–10]. That is, when flight occurs, the co-movement degree in the same direction between markets must decrease. Between the stocks and bonds, flight-to-quality from stocks to bonds and flight-from-quality from bonds to stocks are included. The flight contains two situations of the change to negative correlation in crisis from positive correlation before crisis and the more significant negative correlation in crisis period from negative correlation before crisis. Similar to the definition of contagion, it is not flight if the correlation coefficient remains positive although there is a negative change in two markets, such as the situation of decreasing from 0.7 to 0.2. We can call it flight only when simultaneously satisfying both conditions of a negative decrease and a negative final correlation coefficient. Additionally, both flight-to-quality and flight-from-quality own the common characteristic of significant decrease in correlation coefficients between stocks and the bonds; however, the difference is flight-to-quality occurs when the stock market falls or the bond market rises, and flight-from-quality occurs when the bond market falls or the stock market rises.

From the cross-asset perspective, the cross-market risk contagion and cross-market flight are mutually exclusive. If there is a cross-market risk contagion between two different financial assets, there is no possibility of flights; vice versa. However, these assets can coexist in a cross-country view. Additionally, the risk contagion and flight may mutually promote or weaken. Risk contagion may aggravate (weaken) the flight, and flight may also further aggravate (weaken) risk contagion. When stocks in various countries decline simultaneously due to risk contagions, it may lead to the occurrence of flight from stocks to the bonds between these countries. Conversely, the simultaneous flight from bonds to stocks of each country may lead to risk contagion among bonds among these countries.

By using stocks and bonds as the example, we show the definition of cross-market risk contagion and flight in Table 1:

**Table 1:** Definitions of risk contagion and flight between stocks and bonds.

Situations of markets	Positive change of correlations and positive correlation level	Negative change of correlations and negative correlation level
Stocks falling	contagion (falling simultaneously)	Flight-to-quality
Bonds rising	contagion (rising simultaneously)	
Stocks rising	contagion (rising simultaneously)	Flight-from-quality
Bonds falling	contagion (falling simultaneously)	

### 2.2 Modeling

#### 2.2.1 Testing model for cross-market effects for each country

Based on the study by Baur [6], to capture the correlations between stock and bond markets more accurately and particularly, by combining information theory, we introduce transfer entropy to improve the traditional GARCH model, modeling the dynamic symbolic transfer entropy GARCH(1,1) model to study the cross-market effects between the stock and bond markets for each country. The model is as follows:

$$\frac{{\text{T}}_{\text{b}\to \text{s},\text{t}}}{{\text{T}}_{\text{b}\to \text{s},\text{t}}+{\text{T}}_{\text{s}\to \text{b},\text{t}}}{\text{R}}_{\text{b},\text{t}}=\alpha +\beta \frac{{\text{T}}_{\text{s}\to \text{b},\text{t}}}{{\text{T}}_{\text{b}\to \text{s},\text{t}}+{\text{T}}_{\text{s}\to \text{b},\text{t}}}{\text{R}}_{\text{s},\text{t}}+\gamma \frac{{\text{T}}_{\text{s}\to \text{b},\text{t}}}{{\text{T}}_{\text{b}\to \text{s},\text{t}}+{\text{T}}_{\text{s}\to \text{b},\text{t}}}{\text{R}}_{\text{s},\text{t}}{\text{D}}_{\text{event},\text{t}}+\gamma^{*}\frac{{\text{T}}_{\text{s}\to \text{b},\text{t}}}{{\text{T}}_{\text{b}\to \text{s},\text{t}}+{\text{T}}_{\text{s}\to \text{b},\text{t}}}{\text{R}}_{\text{s},\text{t}}{\text{D}}^{*}{}_{\text{event},\text{t}}+{\text{e}}_{\text{t}}$$ (1)

where e_t_ obeys $\text{N}(0,\sigma_{\text{t}}^{2})$, ${\text{e}}_{\text{t}}=\omega +\lambda {\text{e}}_{\text{t}-1}^{2}+\rho \sigma_{\text{t}-1}^{2}$, t = 1,2,……T.

In which T_b→s,t_ and T_s→b,t_ denote the transfer entropy from bonds to stocks and from stocks to bonds at time t, respectively. $\frac{{\text{T}}_{\text{b}\to \text{s},\text{i},\text{t}}}{{\text{T}}_{\text{b}\to \text{s},\text{i},\text{t}}+{\text{T}}_{\text{s}\to \text{b},\text{i},\text{t}}}$ and $\frac{{\text{T}}_{\text{s}\to \text{b},\text{i},\text{t}}}{{\text{T}}_{\text{b}\to \text{s},\text{i},\text{t}}+{\text{T}}_{\text{s}\to \text{b},\text{i},\text{t}}}$ denote the time-varying impact weights from bonds to stocks and from stocks to bonds at time t, respectively. R_b,t_ and R_s,t_ denote the returns of the bond index and the representative stock index at time t, respectively. β denotes the correlation coefficient of the stocks and bonds in the benchmark period; γ denotes the change of the correlation coefficient between the two markets in the event period, and γ* denotes the correlation coefficient between the two markets before the event. Both D_event,t_ and D*_event,t_ are dummy variables. If t is in the event period, D_event,t_ is 1; otherwise, it is zero. If t before the event, D*_event,t_ is 1; otherwise, it is zero. e_t_ denotes the model error item, complying with the GARCH (1, 1) process.

Considering that there are often fierce fluctuations during a crisis period or event period and that the GARCH model may be able to largely eliminate the change in market correlations caused by the increased fluctuation, we can obtain the change in correlations between markets that is not caused by fluctuation. Conversely, if the dummy variable D*_event,t_ is not added into the model, it means that the correlation coefficient between the stock market and the bond market represented by β during the whole sample is a constant. However, if the correlation is time varying and fluctuates around zero, it does not satisfy the assumption, i.e., not a constant; therefore, it is not suitable to use the whole sample as the benchmark period. Therefore, in addition to the introduction of the dummy variable into the event, we introduce the dummy variable before the event period. In this situation, the benchmark period is not based on the whole sample period but only on the event period and the before event period, which does not include the after-event period; this can cause the model to capture the correlations between markets by more accurate means. Furthermore, considering that the financial market is a complicated system, economic crisis, emergency and black swan events all probably lead to the instability of internal factors in the system and then cause a change in the mutual effects between factors. This change will inevitably lead to influence on the correlation between markets, which will inevitably generate an impact on the correlations of the markets. Therefore, the change information with interactive influence among the internal factors of the financial system has important significance on the estimation of the more objective and reasonable correlation between markets. We utilize the advantage of the transfer entropy, which can capture the statistical correlations that originated from the source sequences. We introduce the impact weights constructed by time-varying symbolic transfer entropy between the stock market and the bond market into the model to capture the interactive influences of internal factors between the stock and the bond markets dynamically. We then estimate the correlation between financial markets more accurately and reasonably.

According to the definition of the cross-market effects in Section 2.1, the cross-market effects between the stock and the bond markets should be determined by both γ and the sum of β and γ. When γ<0 and β+γ<0, there is flight. When γ>0 and β+γ>0, there is risk contagion.

#### 2.2.2 Testing for the simultaneous cross-market effects across countries

Although the model represented by Eq (1) can test the cross-market effects between financial sub-markets, it cannot assess the simultaneity of cross-market effects across countries. Therefore, we construct the symbolic transfer entropy panel GARCH (1, 1) model as follows to test the cross-country effects:

$$\frac{{\text{T}}_{\text{b}\to \text{s},\text{i},\text{t}}}{{\text{T}}_{\text{b}\to \text{s},\text{i},\text{t}}+{\text{T}}_{\text{s}\to \text{b},\text{i},\text{t}}}{\text{R}}_{\text{b},\text{i},\text{t}}=\alpha +\beta \frac{{\text{T}}_{\text{s}\to \text{b},\text{i},\text{t}}}{{\text{T}}_{\text{b}\to \text{s},\text{i},\text{t}}+{\text{T}}_{\text{s}\to \text{b},\text{i},\text{t}}}{\text{R}}_{\text{s},\text{i},\text{t}}+\gamma \frac{{\text{T}}_{\text{s}\to \text{b},\text{i},\text{t}}}{{\text{T}}_{\text{b}\to \text{s},\text{i},\text{t}}+{\text{T}}_{\text{s}\to \text{b},\text{i},\text{t}}}{\text{R}}_{\text{s},\text{i},\text{t}}{\text{D}}_{\text{event},\text{t}}+\gamma^{*}\frac{{\text{T}}_{\text{s}\to \text{b},\text{i},\text{t}}}{{\text{T}}_{\text{b}\to \text{s},\text{i},\text{t}}+{\text{T}}_{\text{s}\to \text{b},\text{i},\text{t}}}{\text{R}}_{\text{s},\text{t}}{\text{D}}^{*}{}_{\text{event},\text{t}}+{\text{e}}_{\text{i},\text{t}}$$ (2)

where i denotes the country. The meanings of the remaining subscripts are the same as that in Eq (1). If γ is significantly different from 0 and if the sum of β+γ has the same symbol as γ, i.e., β+γ>0 and γ>0 or β+γ<0 and γ<0, it is believed that there are cross-market effects between the stock and the bond markets across all countries during the sample period (flight-to-quality, flight-from-quality or risk contagion). That is, the cross-market effects occur simultaneously across all countries. The simultaneity also indicates that the event is the common and fundamental reason leading to simultaneous change in the correlations between the stock and the bond markets of countries.

The maximum likelihood estimation logarithm function in this article is:

$$\text{ln L }(\Psi )=-\frac{\text{T}}{2}\text{ln}(2\pi )-\frac{1}{2}\sum_{\text{t}=1}^{\text{T}}\text{ln}\sigma_{\text{t}}^{\text{2}}-\sum_{\text{t}=1}^{\text{T}}\frac{\left[{\text{W}}_{\text{b}\to \text{s},\text{t}}{\text{R}}_{\text{b},\text{t}}-\text{α}-{\text{W}}_{\text{s}\to \text{b},\text{t}}({\text{βR}}_{\text{s},\text{t}}+\gamma {\text{R}}_{\text{s},\text{t}}{\text{D}}_{\text{event},\text{t}}+\gamma^{*}{\text{R}}_{\text{s},\text{t}}{\text{D}}^{*}{}_{\text{event},\text{t}})\right]}{\text{2}\sigma_{\text{t}}^{\text{2}}}$$ (3)

where ${\text{W}}_{\text{b}\to \text{s},\text{t}}=\frac{{\text{T}}_{\text{b}\to \text{s},\text{t}}}{{\text{T}}_{\text{b}\to \text{s},\text{t}}+{\text{T}}_{\text{s}\to \text{b},\text{t}}}$, ${\text{W}}_{\text{s}\to \text{b},\text{t}}=\frac{{\text{T}}_{\text{s}\to \text{b},\text{t}}}{{\text{T}}_{\text{b}\to \text{s},\text{t}}+{\text{T}}_{\text{s}\to \text{b},\text{t}}}$. Ψ = (α, β, γ, γ*, ω, λ, ρ) is the parameter vector to be estimated.

### 2.3 Transfer entropy

Shannon first proposed information entropy in 1948. After decades of evolvement, Schreiber introduced transfer entropy in 2000 for the first time [11]. The entropy is expressed as follows:

$${\text{T}}_{\text{J}\to \text{I}}=\sum \text{p}({\text{i}}_{\text{t}+1},{\text{i}}_{\text{t}}^{\left(\text{k}\right)},{\text{j}}_{\text{t}}^{\left(\text{h}\right)})\text{log}\frac{\text{p}({\text{i}}_{\text{t}+1}|{\text{i}}_{\text{t}}^{\left(\text{k}\right)},{\text{j}}_{\text{t}}^{\left(\text{h}\right)})}{\text{p}({\text{i}}_{\text{t}+1}|{\text{i}}_{\text{t}}^{\left(\text{k}\right)})}$$ (4)

where $i_{t}^{\left(k\right)}=(i_{t},\ldots \ldots ,i_{t-k+1})$, $j_{t}^{\left(h\right)}=(j_{t}^{},\ldots ,j_{t-h+1}^{})$. $\text{p}({\text{i}}_{\text{t}+\text{1}},{\text{i}}_{\text{t}}^{\left(\text{k}\right)},{\text{j}}_{\text{t}}^{\left(\text{h}\right)})$ represents the transfer probability from status $\left({\text{i}}_{\text{t}}^{\left(\text{k}\right)},{\text{j}}_{\text{t}}^{\left(\text{h}\right)}\right)$ to status i_t+1_. $\text{p}({\text{i}}_{\text{t}+1}|{\text{i}}_{\text{t}}^{\left(\text{k}\right)},{\text{j}}_{\text{t}}^{\left(\text{h}\right)})$ represents the conditional probability of transferring to status i_t+1_ under the status of $\left({\text{i}}_{\text{t}}^{\left(\text{k}\right)},{\text{j}}_{\text{t}}^{\left(\text{h}\right)}\right)$. The $\text{p}({\text{i}}_{\text{t}+1}|{\text{i}}_{\text{t}}^{\left(\text{k}\right)})$ represents the conditional probability of transferring to status i_t+1_ under status ${\text{i}}_{\text{t}}^{\left(\text{k}\right)}$. Additionally, k = h = 1; i.e., both sequences I and J are the first-order Markov process. The transfer entropy from J to I represents the information of sequence I at t+k included in sequence J, excluding the information at t+k contained in the self-sequence I at t. From the expression, we can observe that T_I→J_≠T_J→I_. And when the transfer entropy from I to J T_I→J_ is larger than from J to I T_J→I_: T_I→J_>T_J→I_, although there is flow of information both from I to J and from J to I, the system I transfers more information to J; i.e., the impacts of I on J is larger than J on I. Therefore, overall, it is known that the final net information between I and J (T_I→J_-T_J→I_) flows from I to J, not J to I, and I is considered the information source. Otherwise, when T_J→I_>T_I→J_, it is known that the final net information between I and J (T_J→I_-T_I→J_) flows from J to I, not I to J, and J is considered the information source. Some scholars have applied it to the study in the financial systems [12–18].

Although the transfer entropy contains a directed information measurement, it demands high compatibility from each parameter. Stanick et al introduced the symbolic transfer entropy (STE) to address this problem [19]. By using static dividing, he defined the symbolic values of data according to their specified segments and solved the compatible problem of the parameters. However, static dividing preserves the dynamic features of the original sequences; it also leads to the loss of partial information. Therefore, we adopt the improved dynamic self-adaption symbolic approach proposed in the literature [20] to alleviate this problem. With the representative stock index returns as an example, the specific steps are as follows:

1. Projecting the original time sequences of stock index returns across countries {r(t): 1 ≤ t ≤ T} in an m-dimensional space, respectively, in the following manner:
  R(t)=[r(t),r(t+τ),‥,r(t+(m−1)τ)] (5)
  T represents the sequence length, and τ is the time delay.
2. Calculating the basic scale (BS) for each m-dimensional vector R(t) in (1) as follows [21]:
  $$\text{BS}(\text{t})=\sqrt{\frac{\sum_{\text{j}=1}^{\text{m}-1}{\left(\text{r}(\text{t}+\text{j})-\text{r}(\text{t}+\text{j}-1)\right)}^{2}}{\text{m}-1}}$$
3. According to α × BS, dynamically symbolizing the m-dimensional vector in step (1) as follows:
  $${\text{S}}_{\text{t}}(\text{t})=\left\{ \begin{array}{l} 0,\;\bar{\text{r}}<{\text{r}}_{\text{t}+\text{k}}\leq \bar{\text{r}}+\alpha \times \text{BS},\\ 1,\;{\text{r}}_{\text{t}+\text{k}}>\bar{\text{r}}+\alpha \times \text{BS},\\ 2,\;\bar{\text{r}}-\alpha \times \text{BS}<{\text{r}}_{\text{t}+\text{k}}\leq \bar{\text{r}},\\ 3,\;{\text{r}}_{\text{t}+\text{k}}\leq \bar{\text{r}}-\alpha \times \text{BS}, \end{array}\right.$$ (7)
  where t = 1,2,……,T-m+1, k = 0,1,……,m-1. $\bar{\text{r}}$ represents the mean of the m-dimensional vector, and BS is the corresponding basic scale. After symbolizing the returns, we can calculate the symbolic transfer entropy among the stocks according to Eq (4). In this paper, by using 50 trading days as a sliding window, we calculate the dynamic symbolic transfer entropy from stocks to bonds and bonds to stocks, respectively, and then construct the time-varying impact weights and introduce these into the model to improve the traditional GARCH model.

## Results and discussion

The data consist of the main daily stock indices of the United States and Canada in North America; China and Japan in Asia; Germany, Britain, France and Italy with the top 4 GDP rankings in the EU member states; and Australia in Oceania: Standard & Poor’s 500 index, Nikkei 225 index, German DAX index, France CAC40 index, FTSE-100 index, Italian index, Shanghai composite index, Australian Standard & Poor’s 200 index and Toronto 300 index. The data also include the 10-year bond daily returns of these countries; all data are measured in local currencies. The sample extends from 1 January 2016 to 28 February 2017, and the time period with a month length from 23 June 2016 to 23 July 2016 is used as the event period for Brexit. Given that there are unavailable data due to different national holidays of each country, to address this problem, by adopting the means of the reference [22], we removed the unavailable trading days of each country and then obtain 239 available trading day data. The data were obtained from Wind Database. All the returns are acquired by the logarithmic first-order difference of the daily closing price, i.e.:

$${\text{R}}_{\text{t}}=(\text{ln}{\text{P}}_{\text{t}}-\text{ln}{\text{P}}_{\text{t}-1})\times 100\%$$ (8)

where R_t_ denotes the return on t day, and P_t_ and P_t-1_ denote the closing prices on t and t-1 days, respectively.

### 3.1 The descriptive statistics of the daily stock and bond indices returns of each country

Table 2 provides the statistical characteristics of daily returns of the main stock indices and 10-year bond indices of each country:

**Table 2:** The descriptive statistics of the daily stock and bond indices returns of each country.

	USA		Japan		German		France		Britain		Italy		China		Australia		Canada	
	S	B	S	B	S	B	S	B	S	B	S	B	S	B	S	B	S	B
Mean	0.00	1.74	-0.00	-0.06	-0.00	0.08	-0.00	0.410	0.00	1.27	-0.00	1.39	-0.00	0.00	-0.00	2.26	0.00	1.18
Std. dev.	0.01	0.18	0.02	0.12	0.01	0.21	0.01	0.216	0.01	0.35	0.02	0.15	0.02	0.00	0.01	0.30	0.01	0.12
Skewness	-0.48	0.31	-0.61	0.83	-0.97	0.71	-1.10	0.455	-0.08	-0.22	-1.55	-0.07	-1.67	-0.03	-0.39	0.06	-0.39	0.23
Kurtosis	5.11	2.82	6.45	4.13	6.74	3.05	9.27	2.633	4.62	1.77	12.15	2.24	9.39	4.31	3.70	1.67	4.52	2.67
J-B	45.84	3.04	113.89	28.77	151.46	14.34	376.26	6.852	22.55	12.19	797.66	4.22	444.61	12.15	9.41	12.70	24.88	2.33

S = stock market; B = bond market.

Table 2 shows that the mean daily returns of stock indices of each country during the sample period are zero. However, the mean of bond indices returns is positive except for China, which is zero, and Japan, which is negative.

### 3.2 Estimation results of mean equation of the symbolic transfer entropy GARCH model

Given that the cross-market effects are judged according to the estimation coefficients of the mean equation; therefore, we only list the estimation results of it and not list the estimation results of the variance equation. Tables 3 and 4 provide the estimation results of the mean equation of the symbolic transfer entropy GARCH model, i.e., Eq (1), proposed in this paper:

**Table 3:** The estimation results of mean equation of the symbolic transfer entropy GARCH (1, 1) model.

	USA			Japan			France			Italy			Canada		
	Coef	S.E.	P	Coef	S.E.	P	Coef	S.E.	P	Coef	S.E.	P	Coef	S.E.	P
W_s→b_r_s_	0.6590	0.0365	0.00	0.4621	0.0348	0.00	0.6088	0.0557	0.00	0.5311	0.0391	0.00	0.1127	0.0595	0.05
W_s→b_r_s_D	-0.7486	0.0170	0.00	-0.9754	0.0365	0.00	-0.8442	0.0249	0.00	-0.8008	0.0170	0.00	-0.6603	0.0546	0.00
W_s→b_r_s_D*	-0.7534	0.0241	0.00	-0.6747	0.0151	0.00	-0.3426	0.0233	0.00	-0.4314	0.0158	0.00	0.2095	0.0334	0.00
Constant	0.2050	0.0244	0.00	0.6640	0.0200	0.00	0.3168	0.0372	0.00	0.2246	0.0275	0.00	0.6264	0.0267	0.00
R^2^	0.78			0.78			0.78			0.83			0.72		
Ll	188.31			243.01			192.43			246.59			157.25		
AIC	-1.89			-2.48			-1.94			-3.40			-1.79		
SC	-1.74			-2.32			-1.79			-3.25			-1.57		
ARCH LM	0.634[0.427]			0.154[0.695]			0.108[0.743]			0.287[0.592]			0.370[0.544]		

Coef: the estimated coefficients of corresponding parameters; S.E.: the standard errors of estimated coefficients; P = P-values, represent the significance level of estimated results; [] contains P-values.

**Table 4:** The estimation results of mean equation of the symbolic transfer entropy GARCH (1, 1) model.

	Australia			China			German			Britain		
	Coef	S.E.	P	Coef	S.E.	P	Coef	S.E.	P	Coef	S.E.	P
W_s→b_r_s_	0.3814	0.0498	0.00	-0.0011	0.0743	0.86	0.8591	0.0678	0.00	0.8443	0.0531	0.00
W_s→b_r_s_D	-0.5842	0.0247	0.00	0.1007	0.0693	0.11	-0.9880	0.0508	0.00	-0.9895	0.0565	0.00
W_s→b_r_s_D*	-0.4224	0.0178	0.00	0.0118	0.0630	0.85	-0.7491	0.0358	0.00	-0.8606	0.0399	0.00
Constant	0.3560	0.0383	0.00	0.5429	0.0223	0.00	0.2536	0.0336	0.00	0.3833	0.0279	0.00
R^2^	0.76			0.45			0.74			0.79		
Ll	212.82			156.31			152.16			129.55		
AIC	-2.16			-1.55			-1.551			-1.27		
SC	-2.00			-1.40			-1.36			-1.12		
ARCH LM	0.123[0.726]			1.119[0.292]			0.112[0.737]			0.706[0.402]		

The meanings of Coef, S.E. and P are the same as in Table 3, and [] contains P-values.

Tables 5 and 6 provide the estimation results of the mean equation of the traditional GARCH model:

**Table 5:** The estimation results of mean equation of the traditional GARCH (1,1) model.

	USA			Japan			France			Italy			Canada		
	Coef	S.E.	P	Coef	S.E.	P	Coef	S.E.	P	Coef	S.E.	P	Coef	S.E.	P
r_s_	0.7533	0.0493	0.00	0.5156	0.0331	0.00	0.5993	0.0437	0.00	0.6613	0.0466	0.00	0.2349	0.0645	0.00
r_s_D	-0.9582	0.0345	0.00	-0.9238	0.0166	0.00	-0.9102	0.0289	0.00	-0.8680	0.0176	0.00	-0.8831	0.0475	0.00
r_s_D*	-0.6561	0.0278	0.00	-0.6940	0.0141	0.00	-0.3476	0.0246	0.00	-0.4864	0.0160	0.00	0.0511	0.0320	0.11
Constant	0.3966	0.0298	0.00	0.6069	0.0193	0.00	0.3771	0.0306	0.00	0.2246	0.0305	0.00	0.6324	0.0314	0.00
R^2^	0.64			0.65			0.63			0.72			0.54		
Ll	152.06			232.51			186.87			233.59			141.31		
AIC	-1.51			-2.36			-1.88			-2.37			-1.40		
SC	-1.36			-2.21			-1.63			-2.22			-1.24		
ARCH LM	0.019[0.889]			0.154[0.695]			0.002[0.968]			0.066[0.798]			0.004[0.953]		

The meanings of Coef, S.E. and P are the same as in Table 3, and [] contains P-values.

**Table 6:** The estimation results of the mean equation of the traditional GARCH (1,1) model.

	Australia			China			Germany			Britain		
	Coef	S.E.	P	Coef	S.E.	P	Coef	S.E.	P	Coef	S.E.	P
r_s_	0.1738	0.0768	0.02	-0.0063	0.0818	0.94	0.9305	0.0581	0.00	0.8779	0.0511	0.00
r_s_D	-0.4244	0.0277	0.00	0.1223	0.0755	0.11	-0.9679	0.0507	0.00	-0.9312	0.0469	0.00
r_s_D*	-0.1506	0.0220	0.00	0.0174	0.0690	0.80	-0.8535	0.0399	0.00	-0.86277	0.0449	0.00
Constant	0.4876	0.0572	0.00	0.5378	0.0240	0.00	0.4256	0.0276	0.00	0.3657	0.0255	0.00
R^2^	0.51			0.31			0.77			0.82		
Ll	146.85			147.46			133.42			128.78		
AIC	-1.46			-1.46			-1.32			-1.24		
SC	-1.30			-1.31			-1.16			-1.14		
ARCH LM	0.142[0.706]			0.909[0.341]			2.783[0.197]			0.118[0.732]		

The meanings of Coef, S.E. and P are the same as in Table 3, and [] contains P-values.

From the p-values of the ARCH LM tests shown in Tables 3, 4, 5 and 6, we can observe that both the ARCH effects of the residuals of the two models have been eliminated. However, compared to the estimation results of the traditional GARCH model in Tables 5 and 6, R^2^ (except for Germany and Britain) and the log likelihood in all the countries obtained from the symbolic transfer entropy GARCH model are increased as shown in Tables 3 and 4. In addition, the AIC and SC (except for Britain) of all the countries are decreased, indicating that the model proposed in this paper can fit the data better and have a more practical applicable value. Therefore, we mainly lay the emphasis of the analysis on the empirical results of the symbolic transfer entropy GARCH model shown in Tables 3 and 4.

According to the definition of the cross-market effects, we can obtain the final cross-market effects of each country from the estimation results of the symbolic transfer entropy GARCH (1,1) model in Tables 3 and 4, as shown in Table 7:

**Table 7:** The cross-market effects between stocks and bonds of each country.

	France	German	Britain	USA	China	Australia	Canada	Japan	Italy
Brexit	FTQ	FTQ	FTQ	FTQ	—	FTQ	FTQ	FTQ	FTQ

FTQ = flight-to-quality from stocks to bonds.

From Tables 3 and 4, we can observe that all the estimation coefficients of China are not significant except for the constant terms. However, the correlation coefficients of the remaining 8 countries between stock and bond markets in the benchmark period are significantly positive at the level of 1% (Canada is 5%). Furthermore, in the Brexit event period, the changes in the correlation coefficients of all the remaining 8 countries between stocks and the bonds are significantly negative at the significance level of 1%. This finding indicates that Britain, as the country with the world’s third largest economy, whose capital, London, is also an important global financial center, the Brexit event initiated by Britain has huge spillover effects on the global economy. Additionally, from the statistical results in Table 7, we can observe that, under the influence of the Brexit event, all the stock and bond markets of each country exhibit the flight-to-quality behavior in accordance with the definition of cross-market effects described in Section 2.1, except for China. China may be because the financial markets in China remain relatively closed and have not totally connected with international markets; therefore, the influence of Brexit on Chinese financial markets is relatively limited and not sufficiently large to cause the occurrence of flights. The impacts on China may mainly exist in the trade and currency fields.

Britain, as the main economy of the EU, has close trade relationships and high economic interests with the EU and is the backbone of the economic development of the EU. Britain has an enormous financial industry, and London, the capital of Britain, is the international financial center. Therefore, Brexit will inevitably cause a fluctuation in the financial markets of Britain and other EU members, which will further lead to emotional panic and increasingly fierce risk avoidance emotions for investors. Then, the capital flows into the bond markets with relatively high security and causes the occurrence of flight-to-quality. As Table 7 shows, in the Brexit event period, nearly all the countries except China experienced the flight-to-quality phenomenon. However, according to the estimation results of the change of coefficients W_s→b_r_s_D in the event period, it can be observed that great differences exist in the negative change degree of the correlations between stocks and bonds. Table 7 shows that Britain, Germany and France of EU have relatively larger negative changes of -0.9895, -0.9880 and -0.9754, respectively. For Britain, in the short term, Brexit will affect investor confidence, consumer confidence and business confidence, greatly impacting the stock market negatively, leading to capital flows into the bond market with higher security. Therefore, in the short term, the impact of Brexit on British stock and bond markets is the strongest; thus, the change of coefficient between them is the largest. For Germany and France, which are the main members of EU, both lose an important trade partner after Britain separates from the EU; however, they also must bear a larger economic responsibility in the EU. In the short term, both will suffer a negative impact. Therefore, the stock markets in Germany and France would also be negatively impacted to a certain degree as Britain has, leading to large negative changes in the correlation coefficients between stocks and bonds. Although the stock markets tended to be stable one week later, the influence of Brexit will not disappear in the medium-and-long-term, because it may lead to a domino effect, which may result in a Brexit-like event for other EU members, cause further fluctuations in the financial markets, and then lead to risk contagion and flights again.

It is worth noting that Japan has a negative change of -0.8442, exceeding Italy, a member of the EU, which has a negative change of -0.8008. This finding may be because Japan, as a country that has an extreme reliance on exports, regards Britain as the main hub connecting to European markets, and it has more than 1300 super-heavy large-scale enterprises in Britain, which ranks second in Europe. The separation from the EU will inevitably influence the financial status of Britain, which would then influence the economics of Japan in the EU markets, and thus may lead to a finance depression of Japan. Therefore, in the short term, investors would rather select bonds with low returns but high security and sell stocks; the Brexit event also has a significant influence on Japan in the short term.

Italy and the US have negative changes of -0.8008 and -0.7486, respectively; Canada and Australia have negative changes of -0.6603 and -0.5842, respectively. As a member country of the EU, although the impact endured by Italy is not as large as that of the main members of the EU, Germany and France, the phenomenon of flight-to-quality also occurs to a large degree. For the USA, flight-to-quality also occurs to a certain degree, although not as large as that among the main members of the EU. This difference may be because the USA has a greater trade position than the UK in the EU and because of the dominating role of the US in the world economy, while Britain has a declining importance in the EU, leading to limited influence in the stock market in the United States. In addition, for Canada and Australia, which are commonwealth member states whose stock markets suffer from the negative impacts of Brexit; both exhibit flight-to-quality, but the degree is not as high as the US. This behavior may be because Brexit leads to a closer relationship between Britain and members of the British Commonwealth such as Australia and Canada, resulting in relatively light impacts.

In general, the uncertainty caused by Brexit has influenced the global economy and finance. Britain suffers from Brexit the most, with the deepest flight to quality, followed by Germany, France and Italy in the EU and Japan. This finding regards Britain as the connecting belt to the European market; all these countries experience the flight-to-quality in some degree. Although Brexit causes relatively small impacts on the US, Canada and Australia, it continues to cause the flight-to-quality phenomenon to a relatively light degree. It can be observed that the spillover effects caused by Brexit have resulted in uncertainty in the global financial markets. However, the final influences of Brexit will be determined by the means adopted by Britain and the EU for the negotiation and the time to complete the progress of breaking away from the EU.

The results of the simultaneity of the cross-market effects for all the countries are acquired according to the panel symbolic transfer entropy GARCH (1, 1) model shown in Eq (2), with the results shown in Table 8:

**Table 8:** The estimation results of the panel symbolic transfer entropy GARCH (1, 1) model.

r_b_	Coef	SE	p-Value	Cross-market effects
r_s_	0.3805	0.0179	0.00	Flight-to-quality from stocks to bonds
r_s_D1	-0.8450	0.0161	0.00	
r_s_D* 1	-0.3921	0.0117	0.00	
Constant	0.4947	0.0098	0.00	

As shown in Table 8, the coefficients in the benchmark period, before the event period and in the event period are statistically significant. We can conclude that the flight-to-quality from stocks to bonds phenomenon simultaneously occur in all the countries during the Brexit event period of Brexit. This behavior further indicates that flight-to-quality is a common feature in crisis periods; this finding is consistent with studies by previous academics, such as Baur et al. [6] who studied the correlation between stocks and bonds of eight global economic entities during financial crisis events, Chan et al. used a general Markov switching model to examine the relationships between the returns of five assets in three different asset classes during a tranquil regime and crisis regime [23], and Brière et al. studied four asset classes in four geographical zones during five types of crisis from 1978 to 2010 [24], all of which show that the flight-to-quality between markets regularly occurs in crisis periods. In addition, the result has important implications for asset allocation and hedging. In crisis periods, investors are likely to seek to hedge the risk of crises and reweight toward bonds to avoid the economic losses caused by crises [23]. This behavior indicates that flight-to-quality in crisis periods can potentially avoid the risk of loss, increase resiliency and maintain the stability of financial markets [6]. Furthermore, this behavior also illustrates that the financial markets are complex systems; each market is not isolated but interacts with each other. Any fluctuations of one market would affect other markets within a country or across countries [25,26]. However, this finding may lead to cross-asset risk contagion because the simultaneity of flight-to-quality from stocks to bonds will inevitably cause the stock markets to decline simultaneously and the bond markets to increase simultaneously; i.e., both the co-movement degree of stocks and that of bonds in all countries increase, which is the necessary condition for risk contagion.

## Conclusion

By utilizing the Brexit background, we combine information theory with spatial econometrics, introducing the time-varying symbolic transfer entropy impact weight into the traditional GARCH model to improve the model in this paper. In addition, we study the cross-market effects caused by Brexit between stocks and bonds of the nine main countries in the world. Furthermore, the empirical results show the following:

1. The time-varying symbolic transfer entropy impact weight introduced into the traditional GARCH model can capture the information of mutual influences between internal factors of the stock and the bond markets of each country dynamically and particularly; then, we can capture the correlations among markets more accurately. Thus, the model accuracy can be improved.
2. By the influence of Brexit, the flight-to-quality from stocks to bonds occur widely among these countries. This behavior reveals the security of the bond market and indicates that flight can reduce the loss risk of investors to a certain extent and then increase the resiliency and stability of the financial markets.
3. The simultaneity of flights in the stocks and bonds in countries has a close relationship with cross-country risk contagion. The simultaneous flight-to-quality from stocks to bonds of each country may cause the stocks to decline simultaneously and the bonds to increase simultaneously for each country. However, the simultaneous flight-from-quality from bonds to stocks of each country may cause the stocks to increase and the bonds to decrease simultaneously for each country. Regardless of the kind of flights, this behavior is likely to induce the occurrence of risk contagion between stocks and bonds in countries to further promote the occurrence of flights. Therefore, when investors invest, they need to carefully select the assets and optimize the investment rationally to minimize the risk of losses. When policymakers formulate economic policies, they should control the situation macroscopically and formulate appropriate and effective economic policies to maintain the stability of financial markets.

## Supporting information

S1 Dataset The main daily stock indices and the 10-year bond daily returns of the US, Canada, China, Japan, Germany, Britain, France, Italy and Australia from 1 January 2016 to 28 February 2017 were obtained from Wind Database. The unavailable data due to different national holidays were deleted for all subsequent analyses.
(XLSX)
